# Did You Know: Erythropoiesis Is Regulated by Changes in Posture

**DOI:** 10.1111/apha.70248

**Published:** 2026-05-14

**Authors:** Oscar B. Mazza, Kasper D. Gejl, Steen Larsen, Carsten Lundby

**Affiliations:** ^1^ Department of Sports Science and Clinical Biomechanics University of Southern Denmark Odense Denmark; ^2^ Department of Biomedical Sciences, Faculty of Health and Medical Sciences University of Copenhagen Copenhagen Denmark; ^3^ Clinical Research Centre Medical University of Bialystok Bialystok Poland

Blood volume (BV) is among the most tightly regulated variables in the human body and the small variations of only a few percent likely reflect the central role of BV in the maintenance of arterial blood pressure [1]. In turn, changes in blood pressure affect BV, mainly through dynamic plasma volume (PV) modulation, where decreased central venous pressure (CVP) triggers renal release of volume‐regulating hormones that promote fluid retention [2, 3, 4, 5], while elevations in CVP facilitate renal fluid loss that reduces PV [1, 6]. The potential for CVP to also regulate red blood cell volume (RBCV), however, remains largely unexplored. Members of our group demonstrated that a reduction in CVP—induced by three hours of head‐up tilting—increases circulating erythropoietin (EPO) levels in healthy individuals [7] suggesting that a sustained reduction in CVP may promote erythropoiesis. Changes in RBCV are slower than alterations in PV and typically occur over the course of weeks [8]. Accordingly, we tested whether sleeping in a tilted head‐up position for five weeks to reduce CVP during the night would facilitate erythropoiesis and expand RBCV. For this purpose, nine males (24.2 ± 5.5 yrs.; 85.4 ± 10.8 kg and 188 ± 7 cm) gave oral and written consent to voluntarily participate in the study. The study was approved by the Ethical Committee of Southern Denmark (project ID: S‐20240009) and was conducted in accordance with the Helsinki declaration. In a randomized and counterbalanced order, all participants completed five weeks of either six‐degree head‐up sleeping (HUS) or horizontal sleeping (CON) in their own homes. Trials were separated by a five‐week wash‐out period. Sleep quality (scale of 1–10, with 10 being high and 1 being low) and nightly time spent in bed were recorded. For the first (Pre) and last night (Post), as well after one week (1 W) of the respective trials, the participants slept in the laboratory on a mattress identical to the one they were assigned during that specific trial. On these days, before rising from bed, resting systolic and diastolic blood pressure was determined by sphygmomanometry (M3, OMRON, Kyoto, Japan), and a morning forearm vein blood sample was obtained. Plasma for these samples was stored at−20°C and later analyzed for EPO concentrations.

Blood volume, intravascular volumes (i.e., RBCV and PV), and total hemoglobin mass were determined by use of the carbon monoxide re‐breathing method (Detalo Health, Hørsholm, Denmark) after the first and last night (Pre and Post) of each five‐week block. Interactions and main effects regarding intravascular volumes, blood pressure, and EPO concentrations were tested using a linear mixed‐effects model, with condition and time as fixed effects, and subject as a random effect. If no interaction was present, the analysis was run again excluding the interaction term. Paired Student's *t*‐tests were used to compare average time spent in bed and sleep quality between HUS and CON.

Time spent in bed was 8:03 h·night^−1^ in HUS and 8:04 h·night^−1^ in CON, with no difference between conditions (*p* = 0.55). Sleep quality scores were 7.01 and 7.06 in HUS and CON, respectively (*p* = 0.82). Also, plasma EPO levels and resting systolic and diastolic blood pressure did not differ between conditions at any time point (*p* = 0.38–0.67).

Over the course of the study, RBCV did however increase from 2885 ± 299 to 2990 ± 277 mL in the HUS condition, with no change observed in CON 2922 ± 313 to 2923 ± 305 mL (mean trial difference: 104 mL [26;181] *p* = 0.009) (Figure 1A). In line with this, total hemoglobin mass also increased from 943 ± 101 g to 979 ± 90 g in the HUS condition, with no change in CON 953 ± 102 g to 953 ± 101 g (mean trial difference: 36 g [9;64] *p* = 0.009) (Figure 1D). In contrast, no differences were observed for changes in PV over the course of the five weeks (HUS: 3570 ± 384 mL to 3729 ± 371 mL; CON 3485 ± 493 mL to 3517 ± 476 mL; mean trial difference: 127 mL [−59;312], *p* = 0.182) (Figure 1B). However, an overall effect of condition was present, with HUS displaying an overall greater PV across both the measurements following the first and last night of sleeping (HUS vs. CON: 149 mL [53;245], *p* = 0.002). Collectively, the increase in RBCV together with the numerical increase in PV resulted in a 4.1% increase in total BV in the HUS condition with no difference in CON (HUS 6456 ± 581 mL to 6719 ± 556 mL; CON 6408 ± 759 to 6441 ± 671; mean trial difference 230 mL [16;445], *p* = 0.035) (Figure 1C). This study hence demonstrates that HUS promotes an increase in RBCV, total hemoglobin mass and total BV. Whether the increase in RBCV results from reduced CVP associated with HUS remains unclear and warrants further investigation.

**FIGURE 1:**
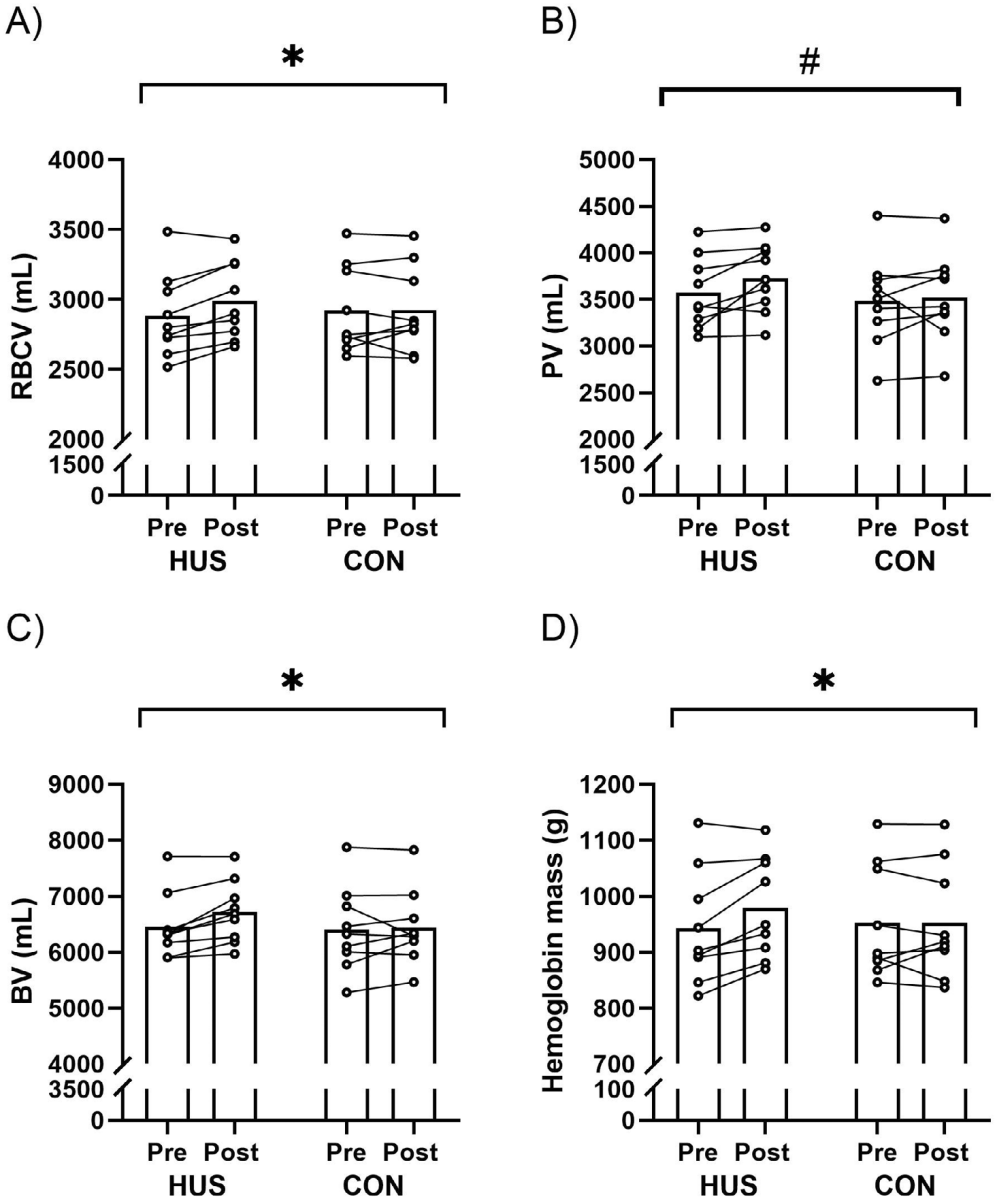
Intravascular volumes, Blood volume, and Hemoglobin mass: (A) red blood cell volume (RBCV), (B) plasma volume (PV), (C) total blood volume (BV), and (D) total hemoglobin mass before (PRE) and following (POST) five weeks of head up sleeping (HUS) or control (CON). Individual participants are connected by lines; * Indicates main effect (condition × time), # indicates overall effect of condition.

One could argue that an increase of 105 mL in RBCV is of limited physiological relevance. However, it should be noted that the intervention period lasted only 5 weeks and that the observed increase in RBCV is comparable to that reported after a similar duration of altitude exposure or exercise training. Ultimately, the study demonstrates that HUS promotes erythropoiesis. From a clinical perspective, intermittent head‐up tilting may be of interest for certain patients subjected to prolonged bed rest as a potential strategy to counteract the associated loss of blood volume, or as an adjunct treatment for patients with anemia. Patients susceptible to development of thrombosis should likely not be included in such attempts.

## Author Contributions

**Steen Larsen:** conceptualization, resources, writing – review and editing. **Carsten Lundby:** conceptualization, funding acquisition, writing – original draft, writing – review and editing, supervision, project administration, resources. **Kasper D. Gejl:** conceptualization, writing – review and editing, supervision, visualization. **Oscar B. Mazza:** writing – original draft, writing – review and editing, conceptualization, investigation, data curation, formal analysis, visualization.

## Funding

This work was supported by a general grant from the Sylvan Adams Sports Institute (Tel Aviv, Israel) to C.L.

## Conflicts of Interest

None of the authors have any conflicts of interest to declare. C.L. is the Founder and CEO of Detalo Health.

## Data Availability

The data that support the findings of this study are available from the corresponding author upon reasonable request.
